# Optimized Hot Pressing of High-Speed Steel–Bronze Composites for Diamond-Reinforced Tool Applications

**DOI:** 10.3390/ma18173999

**Published:** 2025-08-26

**Authors:** Filip Průša, Andrzej Romański, Marzanna Książek, Hana Thürlová, Dorota Tyrała, Petr Kratochvíl, Janusz Konstanty, Ilona Voňavková, František Růžička, Jan Riedl, Robert Dąbrowski, Krzysztof Sołek, Jan Pokorný, Lucyna Jaworska

**Affiliations:** 1 Department of Metals and Corrosion Engineering, University of Chemistry and Technology, Technicka 5, 166 28 Prague 6, Czech Republic; thurlovh@vscht.cz (H.T.); kratochs@vscht.cz (P.K.); vonavkoi@vscht.cz (I.V.); ruzickaq@vscht.cz (F.R.); riedla@vscht.cz (J.R.); pokornyy@vscht.cz (J.P.); 2 Faculty of Metals Engineering and Industrial Computer Science, AGH University of Krakow, Mickiewicza 30 Av., 30-059 Krakow, Poland; aromansk@agh.edu.pl (A.R.); dtyrala@agh.edu.pl (D.T.); konstant@agh.edu.pl (J.K.); rdabrow@agh.edu.pl (R.D.); solek@agh.edu.pl (K.S.); ljaw@agh.edu.pl (L.J.); 3 Faculty of Non-Ferrous Metals, AGH University of Krakow, Mickiewicza 30 Av., 30-059 Krakow, Poland; mksiazek@agh.edu.pl

**Keywords:** ASP60 high-speed steel, hot pressing, bending tests, hardness, porosity, friction coefficients, optimized compaction conditions

## Abstract

This study investigates the optimization of hot-pressing parameters for ASP60 high-speed steel composites incorporating CuSn20 bronze alloy for use in diamond-reinforced tool applications. ASP60 and CuSn20 powders were characterized using XRD, XRF, DSC, SEM, and laser diffraction. The effects of CuSn20 addition at varying concentrations and compaction temperatures (950–1050 °C) on porosity, mechanical properties, and tribological performance were evaluated. Results showed that adding CuSn20 significantly reduced residual porosity due to its partial melting during compaction, which facilitated particle rearrangement and densification. Optimal conditions were identified at 1050 °C with 9.8 wt.% CuSn20, yielding minimal porosity (~3.7%) and the highest bending strength (374.51 ± 36.73 MPa). The optimized matrix was further reinforced with TiC-coated diamond particles at concentration c = 20, producing a composite material with excellent wear resistance, despite minor defects in the TiC coating observed on fracture surfaces. Tribological testing demonstrated that CuSn20 consistently lowered friction coefficients across all tested temperatures due to its self-lubricating properties and partial melting at elevated temperatures. Furthermore, ASP60 exhibited no measurable wear, making it a promising candidate for highly demanding applications. Overall, the study demonstrates that CuSn20 alloy enhances densification, mechanical performance, and tribological behavior of ASP60-based composites, indicating their strong potential for aggressive wire sawing and stone-cutting tool applications.

## 1. Introduction

High-speed steels (HSS) face several challenges when used as matrices for stone-cutting tools, including appropriate wear resistance in highly abrasive stone environments such as granite or basalt, high friction and heat generation leading to softening, and poor thermal conductivity that limits heat dissipation, resulting in tool deformations or loss of diamond retention. Because the diamond grits need to be incorporated at lower temperatures to minimize direct chemical interactions that would otherwise degrade them at higher temperatures, sintering is usually performed at lower temperatures, leading to residual porosity in the steel matrix, which reduces mechanical strength and diamond bonding. To provide continuous and excellent cutting performance, matrixes must exhibit high hardness and yield strength, wear resistance, and retain their properties at elevated temperatures; maintain toughness to resist brittle fracture; ensure good thermal stability; and provide strong diamond retention [1,2,3,4,5,6,7]. Furthermore, low friction or self-lubricating behavior is crucial to reduce heat generation, alongside chemical inertness towards diamond to prevent graphitization and preserve cutting efficiency over prolonged use. Thus, the problems related to high-speed steels are not only exclusively related to the matrix nor to the diamonds but are rather much more complex, and their changes might strongly affect the longevity of such tools. On top of that, the structural homogeneity, porosity, and distribution of diamond particles affect tool performance by altering stress distribution and grit retention under load [5].

ASP60 is considered one of the most promising high-speed steels due to its exceptional properties. It is known for its excellent combination of hardness, wear resistance, and red hardness, making it highly suitable for demanding cutting and tooling applications. Its composition includes high levels of carbon, tungsten, molybdenum, vanadium, and cobalt, providing exceptional hardness (above 65 HRC) and strong resistance to tempering softening. The fine, homogeneous microstructure produced by powder metallurgy ensures superior toughness and dimensional stability compared to conventionally produced high-speed steels. Additionally, its high vanadium content enhances abrasive wear resistance, making ASP60 one of the most wear-resistant steels available, which is crucial for tools operating under severe cutting conditions.

In steel-bonded diamond systems, challenges arise from direct chemical interactions between diamond and iron at sintering or operational temperatures, forming iron carbides instead of maintaining a stable diamond–matrix interface [5,8,9]. This requires adding alloying elements such as titanium, chromium, hafnium, or molybdenum, which form protective carbides (e.g., TiC, Cr_3_C_2_) at the diamond surface, enhancing interfacial bonding and reducing graphitization tendencies [10,11,12,13]. The use of copper- and silver-based brazing fillers in joining diamond segments to steel cores has been investigated to control diffusion and prevent interfacial degradation, where Ti or Zr additions promote carbide formation that improves bonding, while excessive additions can increase graphitization and reduce joint strength [4,10,12,14]. Studies show brazing temperature, filler metal composition, and wetting behavior strongly affect diamond retention and tool life [14].

Diamond, the hardest known material, is nowadays widely used due to its price drop observed over the past three decades in cutting tools due to its superior hardness, thermal conductivity, and chemical stability [5]. However, reinforcing cutting steel and other ferrous materials with diamond tools remains problematic due to severe chemical and thermal degradation mechanisms when hot-pressed at temperatures exceeding 1120 °C, being usually used for high-speed steels [1,2,15]. Thus, the compaction temperature and its decrease in spite of newly developed matrixes are of the utmost importance while also reducing the deleterious porosity [16]. Diamond, being metastable at ambient conditions, transforms to graphite at elevated temperatures, especially in the presence of iron-group metals that catalyze graphitization [6,8,9,17]. This graphitization is detrimental, as graphite is significantly softer, leading to rapid tool wear during steel cutting applications [18].

It is also strongly influenced by the nature of the diamonds, where the natural diamonds start to undergo a transition into graphite at 900 °C while being accompanied by volumetric expansion [19,20], predominating the burning that diamonds experience at lower temperatures [21,22,23,24]. This is different from the synthesized diamonds, which undergo graphitization already at 750 °C. Considering the resistance of diamonds, the oxidation becomes much more severe than the graphitization, since it might develop a large quantity of gases, severely damaging the overall integrity of the composite material [25,26]. The graphitization is being described as a rather complex process composed of several mutual effects composed of the formation of a graphitized surface layer and of graphite nuclei 5–10 nm in size and their further migration towards the diamond surface, creating nucleus nests with aggregated nuclei doubling their sizes [17].

A different but still comparable field of sintered polycrystalline diamond compacts is widely used in applications such as cutting tools and wires or for rock drills. The chosen application strongly influences the lifespan of the working tool, namely by the excessive tool wear due to diffusion processes, cutting resistance, and heat generation, all of which degrade either the tool or the processed material surface while increasing the energy demands [5,27]. The diamond–matrix cohesion could be improved by several approaches, which combine mixing the binder phase with diamonds, surface coatings, or infiltration. In particular, the coating systems of the diamonds have been developed to improve their thermal stability or the chemical bonding with a variety of matrixes. For example, the ZrO_2_-based surface coating reported in [28] allowed the preparation of composite materials to be hot-pressed at extreme temperatures of 1250 °C, far beyond the graphitization onsets for uncovered diamonds. This approach can also enhance the fabrication of diamond-impregnated tools with HSS matrixes, which can then be hot-pressed at higher temperatures, effectively reducing the deleterious porosity of the resulting tools.

Covering the diamonds with Ti or TiC forms a protective barrier acting as an effective inhibitor of oxidation and graphitization, increasing the onsetting temperatures by 50 or 100 °C, respectively [25]. Not only does this suppress both the effects but also protects the diamonds from direct contact with alloys, improving the overall graphitization resistance [6]. On top of that, it possesses excellent mechanical properties, making this technique widely utilized for tool applications. However, thermal stresses due to mismatches in thermal expansion coefficients between diamond (~1 × 10^−6^ K^−1^) and steels or metal binders (~10 × 10^−6^ K^−1^) induce high interfacial stresses under operational temperature fluctuations [5,29]. These stresses can promote the formation of microcracks, edge chipping, and even catastrophic fracture of the diamond grits or the diamond–binder interface [29]. Studies in abrasive and grinding applications show that temperatures exceeding 620–800 °C cause oxidation and graphitization of diamond grains, leading to mechanical degradation and loss of tool efficiency [6,17,22,30]. The fragmentation of the TiC barrier could also be caused by the different coefficients of thermal expansion imposing mechanical stresses, as has been reported in [31], thus promoting the allotropic transformation of diamond into graphite. In fact, TiC possesses one of the highest coefficients of thermal expansion 7.7 × 10^−6^ K^−1^ (1.0 × 10^−6^ K^−1^ for diamond) among the majority of carbides being used for their surface protection [32], causing cracks and further chipping of the TiC as a consequence of stress relief.

Therefore, this study focused on optimized hot-pressing parameters to overcome the long-standing limitations of high-speed steel matrices in diamond-reinforced tool applications. By systematically addressing issues of porosity, diamond degradation, and mechanical performance, this work seeks to establish a promising material system capable of enhancing tool lifespan and cutting efficiency in highly demanding abrasive environments. For this purpose, a commercially available high-speed steel, designated as ASP60, was used to prepare a composite material, with CuSn20 brass (wt.%) added to reduce deleterious porosity.

## 2. Materials and Methods

To optimize the compaction process and enhance mechanical properties while minimizing porosity, ASP60 high-speed steel (Scientific Metal Powders Ltd., Abbeydale Works, Woodseats Road, Sheffield, UK) and CuSn20 bronze alloy (ECKA Granules Germany GmbH, Velden, Germany) powders were selected as base materials. The powders underwent detailed characterization using X-ray diffraction (XRD PANanalytical X’Pert PRO, PANanalytical, Almelo, Holland) for phase analysis and X-ray fluorescence (XRF ARL 9400 XP, Thermo ARL, Écublens, Switzerland) for chemical composition determination.

Rheological properties were assessed by measuring both the tap and apparent densities of the powders. Each measurement was repeated a minimum of three times to ensure statistical reliability. Particle size distribution was determined using laser diffraction analysis with a Malvern Mastersizer 3000+ system (Malvern Panalytical, Almelo, Holland) operating in the wet measurement mode with a dual-laser setup.

Phase transformations within the powders were investigated using differential scanning calorimetry (DSC, TG-DSC Sensys Evo, Setaram, Caluire, France). DSC measurements were conducted in Al_2_O_3_ crucibles under an Ar atmosphere, with a constant heating rate of 10 °C/min. The maximum temperature was set at 1200 °C for ASP60 and 900 °C for CuSn20 to capture the anticipated phase transformations within the alloys during composite alloy compaction.

The water-atomized ASP60 powders were consolidated using a hot press (UNIDIAMOND DC HP, Piacenza, Italy) to produce cuboidal samples. Each sample had a fixed length of 40 mm and a rectangular cross-section with one constant side of 12.2 mm; the other dimension varied with compaction temperature and resulting porosity. For each compaction cycle, 30 g of powder yielded four samples. The compaction process was performed at three different temperatures: 950 °C, 1000 °C, and 1050 °C. The pressure was applied in stages as follows:

- Up to 750 °C: 15 MPa
- Above 750 °C: increased to 20 MPa
- At final temperatures (950/1000/1050 °C): increased to 35 MPa

Porosity of the prepared specimens was quantified using the Archimedes method. To reduce residual porosity, CuSn20 alloy was introduced in the next step into the ASP60 matrix during the compaction. For samples hot-pressed at 950 °C and 1000 °C, 14 wt.% CuSn20 was added, while 9.8 wt.% was used for samples processed at 1050 °C. The content of the CuSn20 alloy was calculated based on the determined porosity of the hot-pressed ASP60 specimens. The optimized compaction condition—ASP60 + 9.8 wt.% CuSn20 at 1050 °C—was used to fabricate a diamond-reinforced composite. Synthetic diamond grits (Hyperion MBS–970, Hyperion Materials & Technologies, Worthington, Ohio, USA, 60/80 mesh, Ti_2_ standing for TiC-coated diamond grits) with a concentration of c = 20 (1 carat cm^3^) were used for reinforcement of the ASP60 steel. These diamond particles were commercially coated with a thick TiC layer to enhance thermal resistance and bonding during the compaction as well as further withstand applications such as aggressive wire sawing.

Mechanical performance of the hot-pressed specimens, including the ASP60, ASP60 + CuSn20, and ASP60 + CuSn20 impregnated with TiC-covered diamond grits, was assessed via three-point bending tests. Samples were sectioned longitudinally for testing at YAW-300G (Jinan Kason Testing Equipment Co., Ltd., Jinan, China) flexural testing machine equipped with a 10kN sensor for accurate force-displacement acquisitions. Fractographic analysis was carried out using scanning electron microscopy (SEM) (Tescan LYRA, Brno, Czech Republic), equipped with Energy-Dispersive Spectroscopy (EDS) (Oxford Instruments, Abingdon, UK, 80 mm^2^ detector).

The tribological behavior of ASP60 and ASP60 + CuSn20 specimens (both hot-pressed at 1050 °C) was examined using a ball-on-disc setup (ELBIT, Koszyce Małe, Poland). Tests employed an Al_2_O_3_ ball under a 5 N normal load, with a sliding speed of 0.5 mm/s over a 5 m total sliding distance. Temperature-dependent wear behavior was studied at 25 °C, 300 °C, 500 °C, and 800 °C.

## 3. Results and Discussion

### 3.1. Powder Characterization

The ASP60 and CuSn20 alloys (all compositions given in weight percent) were characterized using a combination of analytical techniques, including X-ray fluorescence spectroscopy (XRF), X-ray diffraction (XRD), and differential scanning calorimetry (DSC). The elemental compositions determined by XRF are summarized in Table 1 for ASP60 and Table 2 for CuSn20.

In the case of ASP60 high-speed steel, the experimentally determined chemical composition showed a good overall agreement with standard nominal values. However, a noticeable deviation was observed in the concentrations of vanadium (V) and tungsten (W), where the measured contents were 8.250 wt.% and 7.270 wt.%, respectively. These values exceed the typical nominal specification of approximately 6.50 wt.% for both elements. Such enrichment may influence the formation and distribution of secondary carbides, potentially affecting the alloy’s hardness, wear resistance, and response to heat treatment. Relatively high content of Co within the ASP60 might become a problem, as Co acts as a graphitization catalyzer resulting in the chipping and microcracking of the diamond grits, thus increasing the importance of further protective barriers.

The CuSn20 alloy composition remained within the expected compositional range for tin bronzes, with no significant deviations from standard specifications. The collected data serve as the baseline for understanding the subsequent structural and thermal analyses of these alloys.

In contrast, the CuSn20 alloy, which was employed as a porosity-reducing additive in the composite formulation, exhibited only minimal contamination (Table 2). Trace amounts of silicon (Si) and phosphorus (P) were detected, with a combined concentration not exceeding 0.1 wt.%. Such low impurity levels are unlikely to significantly influence the alloy’s metallurgical behavior or its role in enhancing densification during the compaction process.

**Figure 1:**
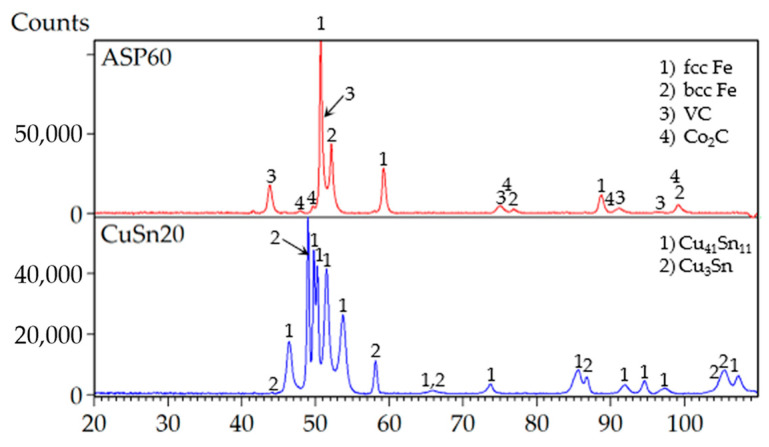
XRD patterns of the powders used for compaction optimization.

X-ray diffraction (XRD) phase analysis (Figure 1) of the powder confirmed the presence of both α-Fe (ferrite, JCPDS card no. 04-002-1253) and retained γ-Fe (austenite, JCPDS card no. 01-074-5520) in the ASP60 alloy. Quantitative evaluation using the Rietveld refinement method revealed that α-Fe constituted approximately 25 wt.%, while retained γ-Fe accounted for 46 wt.% of the crystalline phases. In addition, vanadium carbide (VC) was identified (JCPDS card no. 04-001-2752), comprising 27 wt.% of the sample, along with a minor amount of cobalt carbide (Co_2_C) (JCPDS card no. 03-065-8206).

The CuSn20 powder alloy exhibited a two-phase microstructure consisting of the δ-phase (Cu_41_Sn_11_) (JCPDS card no. 03-065-7047) and the ε-phase (Cu_3_Sn) (JCPDS card no. 04-001-2885). The presence of these intermetallic phases suggests that the original powder may have been produced under high cooling rates, typically achieved by gas atomization methods. These phases are typical in tin bronzes subjected to non-equilibrium solidification, and their coexistence may influence both thermal and mechanical behavior during composite fabrication.

Given that the ASP60 high-speed steel powder was produced via water atomization, it is reasonable to assume that the powder particles possessed oxidized surface layers upon fabrication. During high-temperature compaction, these surface oxides may undergo a thermally activated reduction reaction with carbon inherently present in the steel matrix. Such a reaction could lead to a reduction in oxygen content within the material, potentially enhancing its densification behavior.

To investigate this possibility, differential scanning calorimetry (DSC) analysis was performed on both ASP60 and CuSn20 alloys. For ASP60, a repeated heating cycle was conducted to determine whether any thermally induced reactions were irreversible and associated with oxide reduction.

**Figure 2:**
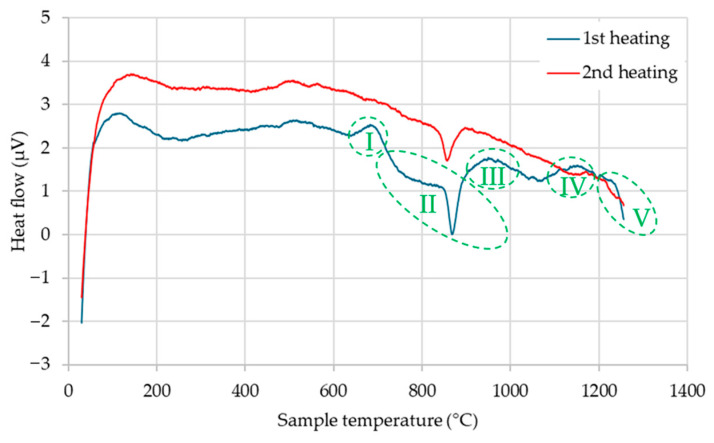
DSC curves of the ASP60 high-speed steel showing the first (*marked with regions I–V*) and consequential second heating of the same sample.

As shown in Figure 2, the DSC thermogram obtained during the first heating of ASP60 reveals five distinct regions, labeled I–V. Regions I, III, and IV exhibit exothermic peaks initiating at approximately 650 °C, 900 °C, and 1100 °C, respectively. These peaks are indicative of chemical reactions, most likely corresponding to the reduction in various surface oxides by carbon present within the steel matrix. This interpretation is supported by the absence of these exothermic features in the second heating cycle (Figure 2), confirming the irreversible nature of these events and strengthening the hypothesis that oxide reduction occurs predominantly during the initial thermal exposure. Similar observations reported in [33] further support the effectiveness of a two-step heating approach in reducing oxide content in the powders, which is beneficial for minimizing diamond degradation via suppressed carbothermal reduction.

Below this temperature range, two additional endothermic regions, designated as II and V, are identified. Region II, starting at approximately 700 °C and extending up to 860 °C, corresponds to the onset of the martensite-to-austenite transformation, reaching its maximum transformation rate around 860 °C. In Region V, at temperatures exceeding 1200 °C, the dissolution of existing carbides begins. These carbides, identified by subsequent XRD analysis as V_8_C_7_ and Fe_3_W_3_C, are thermally destabilized at this stage, leading to their progressive dissolution into the austenitic matrix.

The observed reduction process suggests a concurrent depletion of carbon, which may cause a slight alteration in the steel’s chemical composition. However, the removal of surface oxides could be beneficial, as it enhances the compactability and sinterability of the alloy by reducing interparticle barriers and promoting stronger metallurgical bonding during sintering.

The contents of carbon, nitrogen, and oxygen in the ASP60 powders were determined by elemental analysis to evaluate their changes after the DSC analysis. As shown in Table 3, the contents of both carbon and oxygen decreased, with carbon decreasing from 2.21 wt.% to 2.09 wt.% and oxygen decreasing from 0.285 wt.% to 0.120 wt.%. These results confirm the previously mentioned assumption that oxides within the material were reduced by the carbon present in the steel.

**Figure 3:**
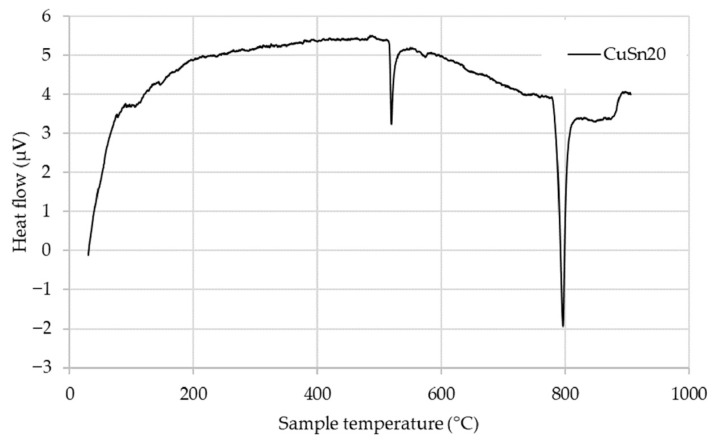
DSC curve of the CuSn20 alloy.

The CuSn20 alloy was also subjected to DSC (Figure 3), which revealed two prominent endothermic peaks associated with thermal phase transformations and melting behavior. The thermal response is consistent with the metastable nature of the CuSn20 alloy, which likely originates from its specific powder production method. Upon heating, the alloy begins to transform toward a more thermodynamically stable phase equilibrium.

Initially, within the temperature range up to approximately 500 °C, the metastable structure gradually evolves into a mixture of α + δ and subsequently α + ε phases. A sharp endothermic peak is observed near this temperature, marking a critical transformation. The first major transformation, beginning slightly above 525 °C, corresponds to the α + δ → α + γ phase transition.

With continued heating, the system undergoes an additional reaction, transitioning from α + γ → α + β, followed by the onset of partial melting, characterized by the reaction α + β → α + L. This partial melting process is responsible for the second significant endothermic peak, occurring at approximately 778 °C, which aligns closely with the reported peritectic temperature for CuSn20 alloys.

Upon further heating above 930 °C, complete melting of the alloy occurs, in agreement with previously published data [34,35]. These observations are consistent with the findings in [36], reporting two distinct endothermic peaks during DSC analysis of Cu–Sn-based alloys, confirming the reproducibility of this thermal behavior.

**Figure 4:**
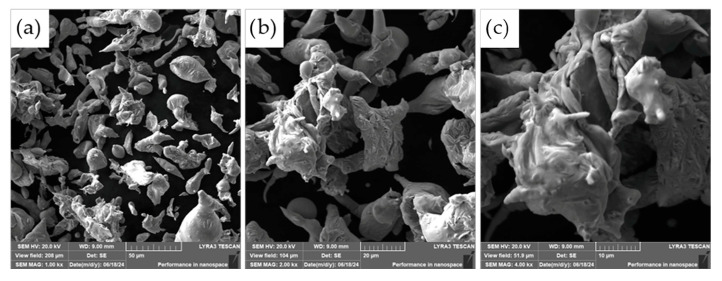
Morphology of the ASP60 powder showing various magnifications (SEM, 20 kV, SE detector). (**a**) 1000×; (**b**) 2000×; (**c**) 4000× magnifications.

The morphology of the powder particles was further examined using scanning electron microscopy (SEM), as presented in Figure 4. The observed microstructure of the ASP60 powders is consistent with the characteristics typically associated with the water atomization process. The particles exhibit predominantly irregular and rounded shapes, with a portion of larger particles displaying significant flattening. This deformation is likely a result of interactions between molten droplets and high-velocity water jets, as well as collisions with the walls of the atomization chamber during solidification.

The resulting morphology is representative of powders produced by water atomization and shares several similarities with powders fabricated via alternative atomization methods, such as gas atomization or centrifugal atomization. However, due to the aqueous environment employed in water atomization, some degree of surface oxidation is anticipated.

**Figure 5:**
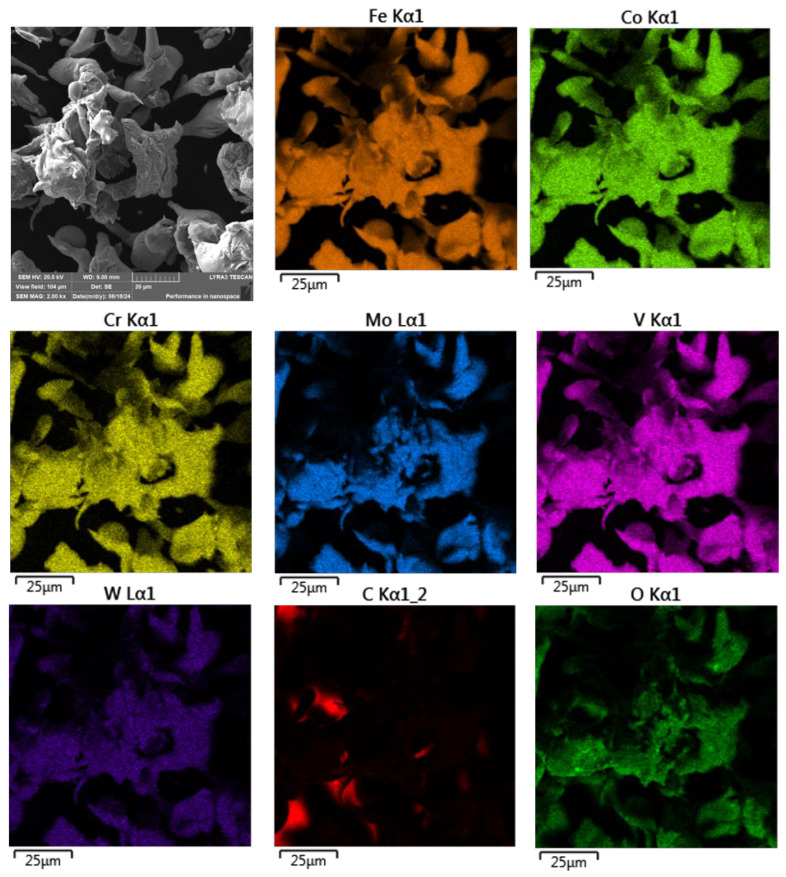
SEM + EDS element distribution maps of the ASP60 water-atomized powder.

To investigate this, SEM analysis coupled with energy-dispersive X-ray spectroscopy (EDS) elemental mapping was employed to assess the distribution of oxidized elements on the particle surfaces. The resulting maps, shown in Figure 5, provide qualitative evidence of oxide formation, particularly at the surface regions of the powder particles.

As illustrated in Figure 5, the ASP60 powder particles exhibited overall good elemental homogeneity with respect to both their primary and alloying constituents. However, surface oxidation was clearly evident on several particles, in agreement with prior assumptions based on the water atomization process.

Although the oxidation appeared generally uniform across most particle surfaces, certain particles exhibited highly localized regions with significantly elevated oxygen concentrations relative to their surroundings. These localized oxidized zones provide further evidence of surface-level oxidation, likely resulting from the rapid solidification and exposure to the aqueous environment during atomization. The presence of such oxidation may influence the powder’s reactivity and sinterability in subsequent high-temperature processing steps.

A similar investigation was conducted on the CuSn20 alloy, which served as both a binder and a porosity-reducing agent in the compaction process. Due to its relatively low melting point and favorable flow characteristics, CuSn20 facilitates the rearrangement of ASP60 high-speed steel particles and aids in filling interstitial voids during hot pressing, thereby enhancing densification.

**Figure 6:**
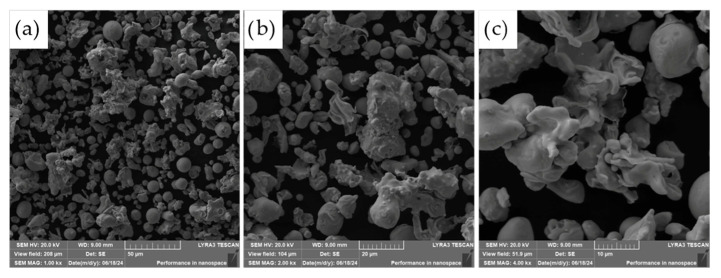
Morphology of the CuSn20 powder at various magnifications (SEM, 20 kV, SE detector). (**a**) 1000×; (**b**) 2000×; (**c**) 4000× magnifications.

The morphology of the CuSn20 powder particles is presented in Figure 6. Compared to the ASP60 powder, the CuSn20 particles are significantly smaller in size and exhibit a predominantly spherical to near-spherical shape. This morphology is indicative of a gas atomization production route, which is commonly used to produce powders with high flowability, narrow particle size distributions, and minimal surface oxidation. The observed characteristics are consistent with the intended role of CuSn20 in promoting uniform particle packing and improved sinterability due to the presence of a liquid phase within the composite system.

**Figure 7:**
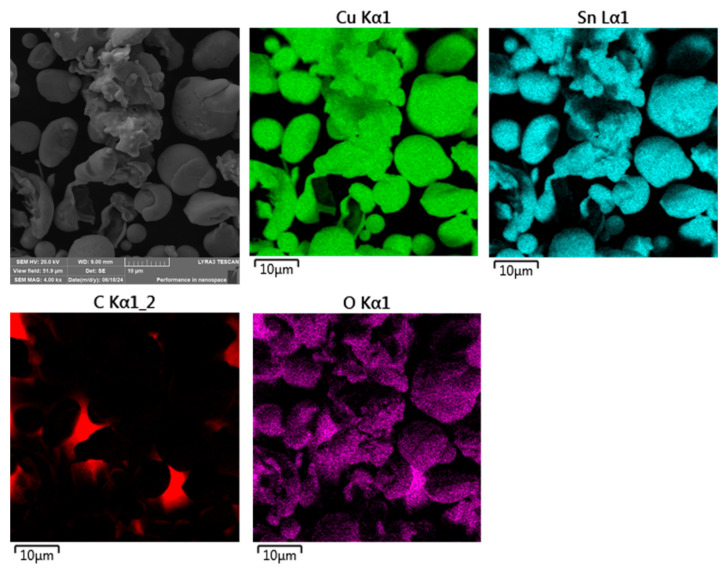
SEM + EDS element distribution maps of the CuSn20 water-atomized powder loosely placed on double-sided adhesive tape.

The CuSn20 powder alloy was further examined for elemental distribution using scanning electron microscopy (SEM) coupled with energy-dispersive X-ray spectroscopy (EDS), as shown in Figure 7. The analysis confirmed a generally homogeneous distribution of the primary elements—copper (Cu) and tin (Sn)—throughout the powder particles. However, localized regions exhibiting elevated oxygen concentrations were also identified on the particle surfaces.

Such surface oxidation is characteristic of fine powders produced by gas atomization, where the high surface-area-to-volume ratio of the particles, combined with residual oxygen in the atomization environment, promotes the formation of surface oxides. These oxidized zones, although limited in extent, may influence the sintering behavior and interfacial bonding characteristics during composite consolidation.

The rheological properties of the ASP60 and CuSn20 powders were evaluated through measurements of their apparent (Table 4) and tap densities (Table 5), as well as particle size distribution. Apparent density measurements revealed relatively low bulk packing densities for both powders, with values of 1.270 ± 0.052 g/cm^3^ for ASP60 and 3.183 ± 0.021 g/cm^3^ for CuSn20. These low values reflect the initial loose packing state of the powders when poured without external compaction.

Subsequent tap density measurements, which involve mechanical vibration or tapping to promote particle settling and reduce interparticle voids, demonstrated a significant increase in packing efficiency. The tap density values increased to 1.949 ± 0.110 g/cm^3^ for ASP60 and 4.225 ± 0.510 g/cm^3^ for CuSn20. This increase reflects the improved particle rearrangement and packing stability under external agitation. Notably, the higher densities observed for CuSn20 in both apparent and tap conditions are consistent with its smaller particle size and spherical morphology, which favor more efficient packing compared to the larger, irregular particles of ASP60.

**Figure 8:**
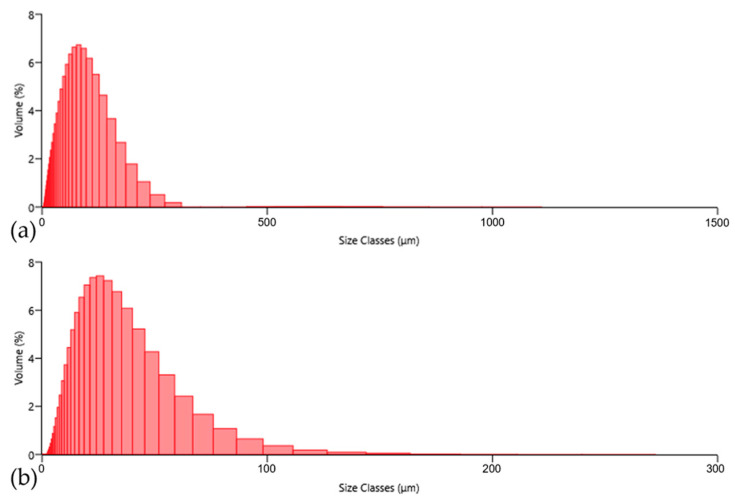
Powder laser diffraction analysis of particle size distributions: (**a**) ASP60; (**b**) CuSn20 alloy.

The particle size distributions of the ASP60 and CuSn20 powders were determined using laser diffraction analysis (Malvern Mastersizer 3000+), showing the histograms of the powder particle distributions in Figure 8. The ASP60 powder exhibited particle sizes ranging from 2.8 to 309.5 μm, with a median diameter (D_50_) of 62.0 μm, indicating a relatively broad particle size distribution. In contrast, the CuSn20 powder consisted of significantly finer particles, with sizes ranging from 1.4 to 211.1 μm and a D_50_ value of 22.8 μm.

Based on these distributions, the specific surface areas of the powders were calculated, yielding 160.6 m^2^/kg for ASP60 and 352.7 m^2^/kg for CuSn20. The markedly higher surface area of the CuSn20 powder reflects its finer particle size and narrower distribution, which can influence its sintering behavior due to a more homogeneous distribution prior to its melting during the hot-pressing compaction.

**Figure 9:**
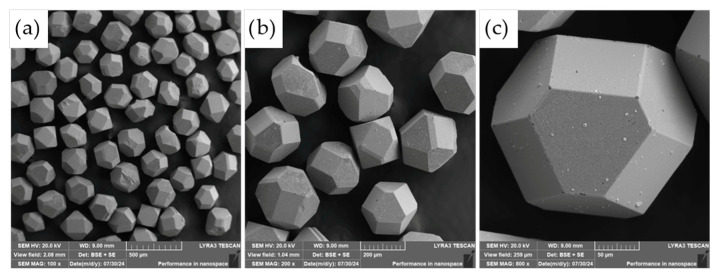
SEM micrographs of diamonds covered with a TiC layer. (**a**) 1000×; (**b**) 2000×; (**c**) 4000× magnifications.

The commercially supplied diamond particles (Hyperion, 60/80 mesh), pre-coated with a titanium carbide (TiC) protective layer, were analyzed in detail using scanning electron microscopy (SEM) combined with energy-dispersive X-ray spectroscopy (EDS). As illustrated in Figure 9, the diamonds exhibit the characteristic angular morphology typical of abrasive and cutting tool applications. The TiC coating serves a critical function as a thermal and chemical barrier, designed to suppress graphitization and chemical degradation of the diamond core during high-temperature compaction processes.

While the majority of the particles displayed a continuous and uniform TiC layer, minor flaws in the coating were observed on a subset of particles, particularly along sharp edges and corners (see Figure 9c). These localized defects may act as vulnerable sites for chemical interaction, mechanical damage, or thermal degradation during sintering, potentially compromising the long-term stability and performance of the composite. Such observations highlight the importance of coating integrity in high-temperature applications where strong matrix–diamond interactions occur.

**Figure 10:**
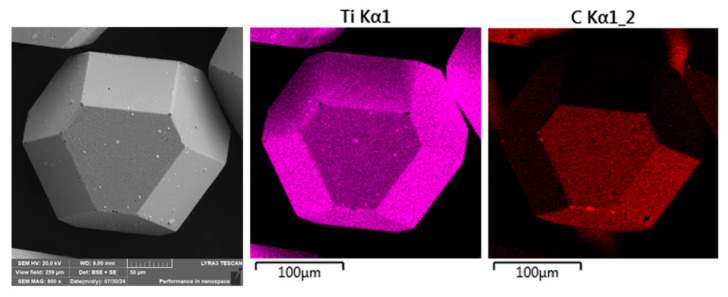
SEM + EDS element distribution maps of TiC-coated diamonds used for the creation of the diamond-reinforced composite.

Detailed SEM–EDS elemental distribution mapping (Figure 10) confirmed the presence of defects in the TiC protective coatings, as previously described. These flaws were observed not only along the sharp edges and corners of the diamond particles but also across some flat surface areas. The observed damage is most likely attributable to the batch-wise handling of the TiC-coated diamonds during storage, transport, or processing. Given the extreme hardness of diamond, individual particles may act as abrasive agents or impact sources, mechanically compromising the integrity of the relatively softer TiC coating.

Such mechanical interactions can result in localized chipping, cracking, or delamination of the TiC layer, particularly at geometrically exposed regions where the coating is more susceptible to stress concentrations. These defects may serve as entry points for diffusion or chemical reactions during high-temperature compaction, potentially undermining the protective function of the TiC barrier and reducing the overall thermal stability of the diamond reinforcements within the composite.

### 3.2. Optimization of Hot Pressing

To evaluate the influence of compaction temperature on residual porosity, uniaxial hot pressing was conducted at three different temperatures: 950 °C, 1000 °C, and 1050 °C. Powder batches of 30 g were loaded into a graphite mold and hot-pressed under a constant pressure of 35 MPa for a dwell time of 3 min at the specified temperatures. This experimental setup was designed to simulate typical conditions for the consolidation of powder-based composites.

**Figure 11:**
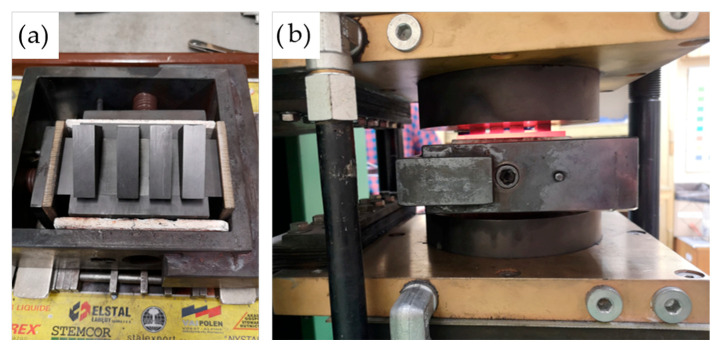
Photodocumentation of (**a**) the mold assembly; (**b**) the compaction process.

The complete mold assembly, hot-pressing apparatus, and compaction process are depicted in Figure 11, illustrating the alignment of the tooling and the thermal–mechanical environment in which densification was achieved. These conditions were selected to assess the effect of increasing compaction temperature on pore elimination, densification behavior, and potential phase evolution during processing.

Following compaction, the prism-shaped specimens were ground using a diamond grinding disc to ensure uniform surface finish and dimensional accuracy. Residual porosity was then determined using two complementary methods: dimensional analysis and the Archimedes method, with the results summarized in Table 6. For the Archimedes measurements, the open porosity was sealed by immersing the samples in molten paraffin wax prior to submersion in water, ensuring a more accurate assessment of closed porosity.

Based on the porosity results obtained from specimens made of pure ASP60 powder, additional mixtures containing ASP60 and CuSn20 were prepared and hot-pressed to evaluate the effect of the alloying additive on densification. As shown in Table 6, the incorporation of CuSn20 led to a notable reduction in residual porosity. This improvement is primarily attributed to the partial melting of the CuSn20 alloy during high-temperature compaction, which facilitates the rearrangement of ASP60 particles and promotes densification by filling interparticle voids. The molten phase acts as a transient liquid binder, enhancing packing efficiency and bonding within the composite structure.

The mechanical performance of the samples was evaluated using three-point bending tests, with the results summarized in Table 7. The highest bending strength—374.51 ± 36.73 MPa—was achieved by the ASP60 + CuSn20 composite hot-pressed at 1050 °C, which also exhibited the lowest residual porosity among all tested compositions. The porosity of this sample was measured at 3.70% by the Archimedes method and 3.69% by dimensional analysis, indicating excellent agreement between the two techniques and highlighting the effectiveness of the densification process at this temperature.

In general, compaction at 1000 °C or higher was found to significantly enhance mechanical properties, regardless of the presence of CuSn20. However, the addition of CuSn20 further amplified these improvements by reducing residual porosity and promoting more efficient particle rearrangement and bonding during hot pressing.

It is important to note that substantial variation in apparent surface porosity was observed among the tested samples. This heterogeneity is attributed to the uniaxial compaction process, where frictional resistance—both between powder particles and between particles and the mold walls—impedes uniform pressure transmission. This results in non-uniform densification, particularly at the sample surfaces.

To account for this effect in mechanical testing, sample orientation was determined via optical microscopy. The more porous surface was deliberately positioned on the tensile side of the bending test setup, representing the most critical area for crack initiation. Consequently, the bending strength values reported in Table 7 should be regarded as conservative lower-bound estimates, strongly influenced by residual porosity.

It is anticipated that substantially higher mechanical performance could be achieved through the use of advanced consolidation techniques, such as hot isostatic pressing (HIP), which can produce near-full-density materials with minimal porosity and enhanced structural integrity.

Based on the obtained results, Vickers hardness (HV30) and Rockwell hardness (HRC) measurements were conducted on both the tensile and compressive sides of the specimens sintered at 1000 °C and 1050 °C. These specific temperatures were selected due to the pronounced differences observed in surface porosity, which warranted further investigation into the localized mechanical response. The hardness data are summarized in Table 8 (Vickers) and Table 9 (Rockwell). Compared to other work [37], the hereby reported samples were showing at least three times higher Vickers hardnesses compared to their low-alloy steel that has been hot-pressed at significantly lower temperatures within a range of 820–950 °C.

The tensile side—corresponding to the surface opposite the upper movable piston during uniaxial compaction—consistently exhibited hardness values with higher relative standard deviations (RSDs) and wider 95% confidence intervals compared to the compressive side. This variability reflects the non-uniform porosity distribution within the specimens, which is significantly influenced by the position within the sample.

Such heterogeneity is a known limitation of uniaxial compaction, where friction between powder particles and the die wall results in uneven force transmission, leading to density gradients across the green body. These gradients persist through sintering and directly impact the mechanical properties at different regions of the compact. Moreover, the observed uneven pressure distribution would further affect the lifespan of diamonds due to the formation of tensile stresses on their surfaces in areas where a pore is present, leading to pressure drops and enhancing the localized graphitization of the diamond surface.

To overcome the limitations associated with uniaxial compaction—particularly the friction-induced anisotropy and resulting inhomogeneous densification—hot isostatic pressing (HIP) presents a highly effective alternative. Unlike uniaxial pressing, HIP applies uniform isostatic pressure in all directions, eliminating density gradients and promoting homogeneous microstructural development throughout the compact. As a result, HIP enables the production of near-full-density materials with minimal residual porosity and consistent mechanical properties across the entire volume. Given these advantages, it is anticipated that the application of HIP would lead to substantially enhanced mechanical performance, making it a superior consolidation technique for structural components requiring high reliability and integrity.

**Figure 12:**
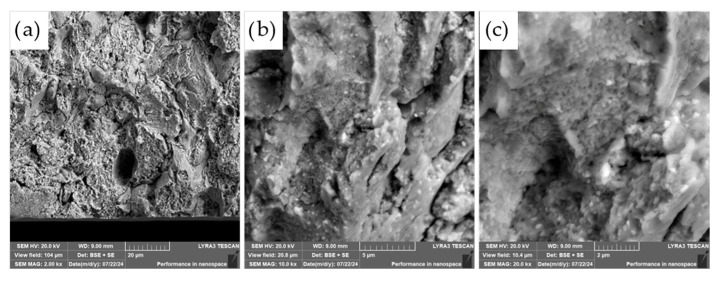
SEM micrographs of the fracture surface of the ASP60 alloy hot-pressed at 1000 °C showing different magnifications in (**a**–**c**) (*tensile site*).

**Figure 13:**
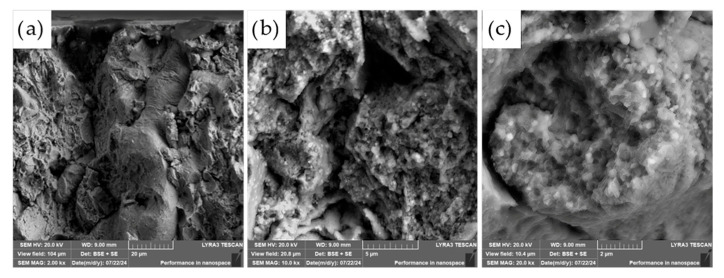
SEM micrographs of the fracture surface of the ASP60 alloy hot-pressed at 1000 °C showing different magnifications in (**a**–**c**) (*compressive site*).

**Figure 14:**
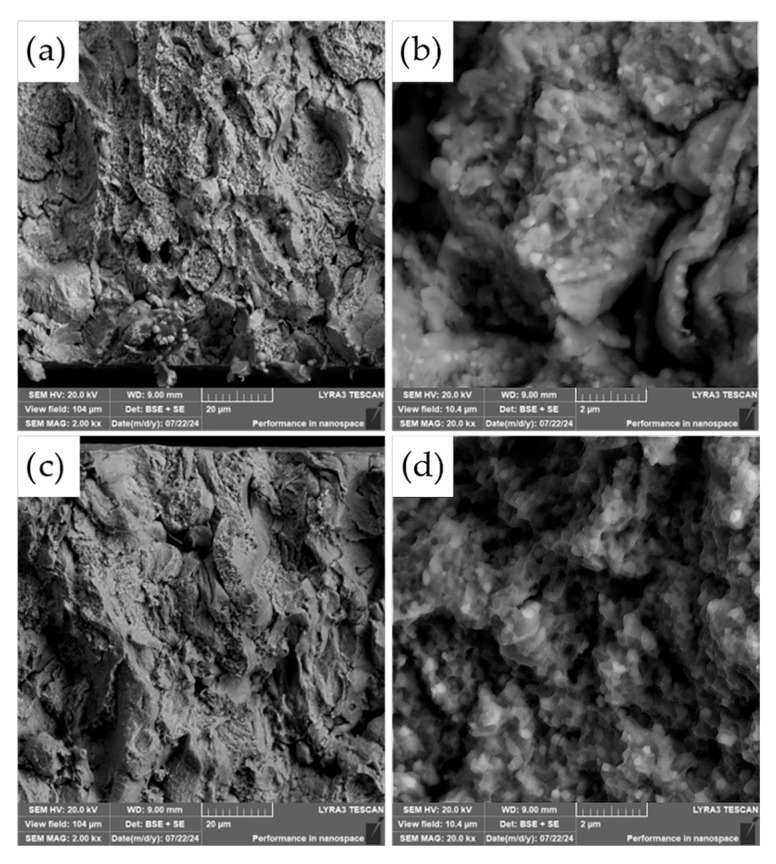
SEM micrographs of the fracture surface of the ASP60 alloy hot-pressed at 1050 °C: (**a**,**b**) tensile site; (**c**,**d**) compressive site.

The fracture surfaces of the most promising specimens—ASP60 and ASP60 + CuSn20 sintered at 1000 °C and 1050 °C—were investigated using scanning electron microscopy (SEM) coupled with energy-dispersive X-ray spectroscopy (EDS). The analysis revealed that the overall fracture morphology remained largely consistent, regardless of the region examined (i.e., the more porous area, as shown in Figure 12, versus the crack initiation site, Figure 13) or the applied compaction temperature (see Figure 14).

The observed fracture surfaces exhibited features characteristic of mixed-mode failure, combining transgranular fracture with quasi-cleavage facets. Additionally, numerous particle pull-outs were evident, leading to the formation of voids, indicative of weak interparticle bonding in localized regions. Notably, the presence of micro-dimples was also observed, which are typically associated with ductile fracture behavior. These dimples likely originate from the plastic deformation of retained austenite regions as well as decohesion around fine carbide particles.

The carbide particles, uniformly distributed across the fracture surface, displayed an average diameter of approximately 200 nm. Their homogeneous dispersion suggests effective particle bonding and consistent microstructural development throughout the sintered matrix. These findings underscore the complex fracture mechanisms at play and highlight the influence of retained austenite and second-phase particles on the overall fracture behavior of the specimens.

**Figure 15:**
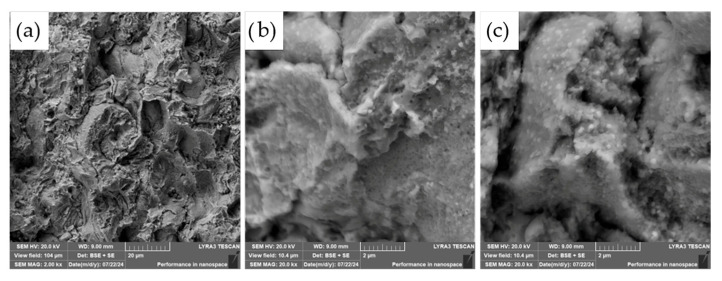
SEM micrographs of the fracture surface of the ASP60 + CuSn20 alloy hot-pressed at 1050 °C: (**a**,**b**) porous site; (**c**) crack site.

However, a notable distinction emerges when comparing the fracture surfaces of the ASP60 alloy with those of the ASP60 + CuSn20 composite. In the latter, distinct regions corresponding to the CuSn20 phase are evident, appearing as brighter areas in the SEM micrographs (Figure 15). These regions indicate the presence of the CuSn20 alloy within the matrix, suggesting a heterogeneous microstructure in which the secondary phase is embedded within the ASP60 framework.

**Figure 16:**
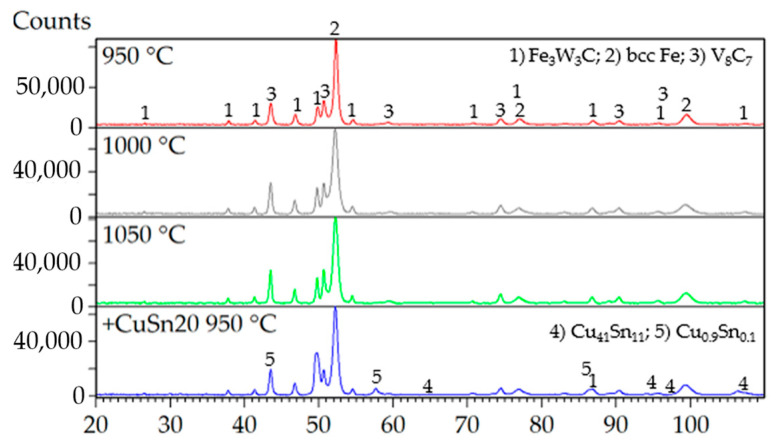
XRD diffraction patterns of ASP60 alloy being hot-pressed at different temperatures as well as of the ASP + CuSn20 composite hot-pressed at 950 °C.

Evaluation of the mechanical properties clearly indicated that porosity is the dominant factor negatively affecting the overall performance of the investigated materials. Despite the variation in compaction temperatures, X-ray diffraction (XRD) analysis revealed that the phase composition of the specimens remained largely invariant (Figure 16). The primary phases identified across all samples included a ternary carbide Fe_3_W_3_C (JCPDS card no. 04-006-1675), a binary vanadium carbide V_8_C_7_ (JCPDS card no. 01-089-2608), and body-centered cubic (BCC) Fe (JCPDS card no. 04-002-1253), corresponding to the presence of martensite. The presence of martensite could be explained by the ability of ASP60 high-speed steel to undergo a phase transformation of retained austenite during plastic deformation, which is introduced, e.g., due to grinding.

The addition of 9.8 wt.% CuSn20 alloy to the ASP60 matrix significantly reduced porosity and led to the formation of additional intermetallic phases. The majority of these phases corresponded to Cu_0.9_Sn_0.1_ (JCPDS card no. 04-018-6729), while a smaller fraction, amounting to several weight percent, was identified as Cu_41_Sn_11_ (JCPDS card no. 01-071-0094). The observed BCC Fe phase may be associated with low-carbon martensitic structures or other ferritic constituents, which are not easily distinguishable by conventional methods such as optical microscopy (OM) or scanning electron microscopy (SEM).

**Figure 17:**
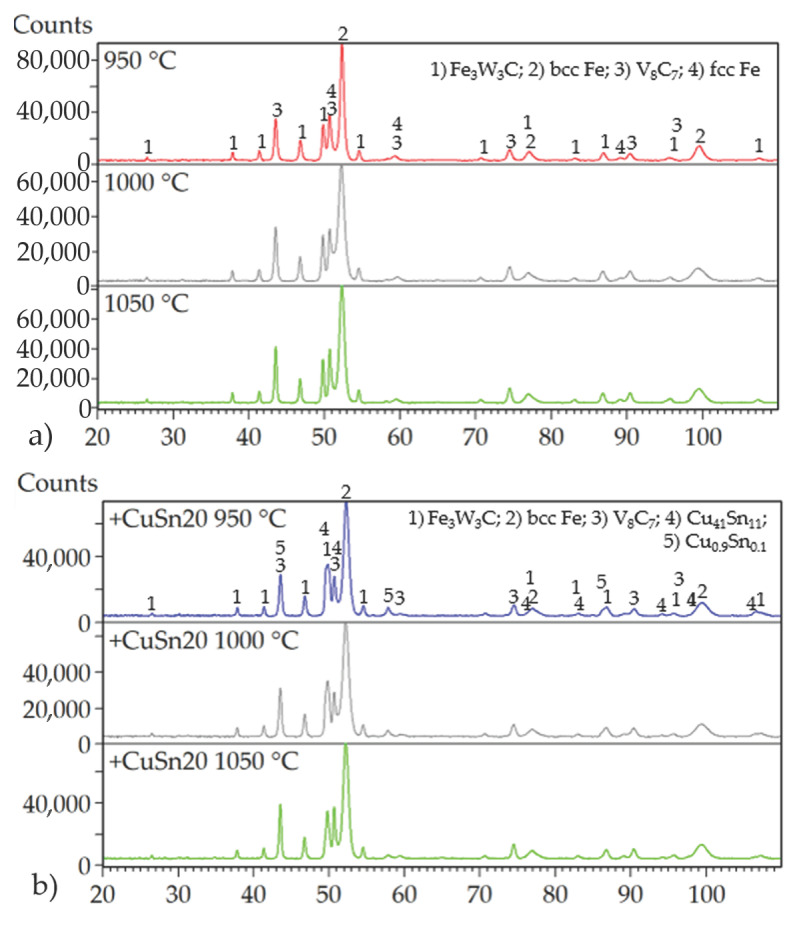
XRD diffraction patterns of (**a**) ASP60 alloy; (**b**) ASP + CuSn20 alloy after deep etching.

Nevertheless, upon deep etching, all the specimens revealed the presence of small fractions of retained austenite (Figure 17) characterized by a face-centered cubic (FCC) crystal lattice. This observation suggests that some metastable austenitic regions were retained during cooling, potentially contributing to localized ductility within the otherwise hard matrix. Except for exposing the retained austenite, the phase composition was identical to that observed in the case of prepared specimens without deep etching.

Based on these findings, a compaction temperature of 1050 °C combined with the addition of 9.8 wt.% CuSn20 was identified as the most promising processing condition for the fabrication of a composite material reinforced with TiC-coated diamond particles. This combination offers an optimal balance between densification, phase stability, and microstructural uniformity.

### 3.3. Properties of Composite Material (ASP60 + CuSn20 + TiC/Diamond)

The diamond-reinforced composite material, comprising ASP60 high-speed steel, 9.8 wt.% CuSn20 bronze, and TiC-coated diamond particles at a concentration of c = 20, was fabricated via hot pressing at 1050 °C. The compaction process was carried out under uniaxial pressure with a constant dwell time of 3 min. The total porosity of the resulting composite was evaluated using dimensional analysis, yielding an approximate value of 9.16%. It should be noted that this measurement is influenced by the altered effective density of the multiphase system, which was assumed, according to the ASP60 product list [38], to be 8.0 g/cm^3^, consistent with previous experimental conditions.

As summarized in Table 10, the flexural strength (359.1 ± 26.5 MPa) of the diamond-reinforced composite is comparable to that of the ASP60 + CuSn20 matrix material (374.51 ± 36.73 MPa, see Table 7). However, despite the similar mechanical strength, the composite exhibits significantly enhanced tribological behavior. The material demonstrated extreme resistance to abrasive wear, to the extent that it could barely be machined using diamond grinding plates. This indicates a substantial improvement in wear resistance and underscores the potential of this composite for application in highly demanding tribological environments.

**Figure 18:**
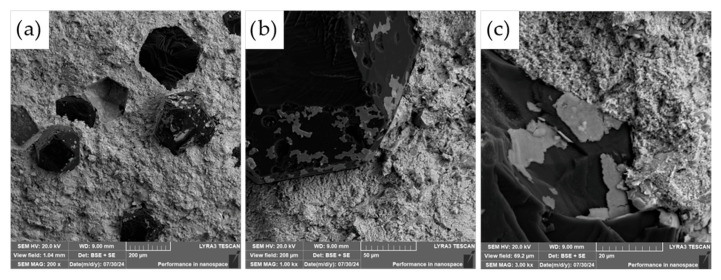
SEM micrographs of the fracture surface of the ASP60 + CuSn20 + 20TiC/diamond composite alloy hot-pressed at 1050 °C, showing (**a**) an overview; (**b**) a diamond with some surface damage; and (**c**) a detailed view of the remaining TiC surface layer.

The fracture surface was also investigated in more detail using SEM and EDS, as shown in Figure 18. 

As shown in Figure 18, examination of the fracture surfaces following flexural testing revealed the presence of diamond particles with partially preserved TiC coatings. In many cases, the absence of the TiC layer in the fracture zone suggests good interfacial cohesion between the protective coating and the ASP60 high-speed steel matrix, implying that the coating remained well-bonded during both processing and mechanical loading.

Closer inspection (Figure 18b) shows that some diamond particles exhibit surface damage in the form of graphitization pits and edge chipping similar to the work of Shevchenko et al. [23]. These features are most likely associated with regions where the TiC coating was either absent or degraded, as previously indicated in Figure 9 and Figure 10. During hot pressing, such uncoated diamond surfaces come into direct contact with the metallic matrix, which can result in localized chemical interactions and mechanical interlocking. This may enhance bonding strength but also increases the risk of undesired reactions at elevated temperatures, such as surface graphitization of diamond grits.

At compaction temperatures exceeding 800 °C—particularly at the 1050 °C temperature used in this study—the risk of diamond degradation becomes significant. Direct contact between diamond and the iron-based matrix can lead to carbon dissolution into the steel or the onset of diamond graphitization. In this context, the TiC coating serves not only as a physical interface but also as a critical thermal and chemical barrier. Its integrity is essential for suppressing high-temperature reactions that compromise the structural stability of the diamond reinforcements. Therefore, the effectiveness and uniformity of the TiC coating are key to maintaining both the mechanical integrity and tribological performance of the composite.

## 4. Tribological Properties

The metallic matrices used in segments for stone-cutting tools must exhibit a low coefficient of friction to help maintain relatively low operating temperatures during tool use. This thermal management is critical to preventing thermal damage to both the matrix and the diamond particles. However, unlike in conventional wear-resistant applications, minimal matrix wear is not a priority in this context. On the contrary, a controlled wear rate of the matrix is desirable, as it facilitates the progressive exposure of new diamond cutting edges. Additionally, effective cohesion between the diamonds and the matrix is essential to ensure the stability of the abrasive elements during operation.

**Figure 19:**
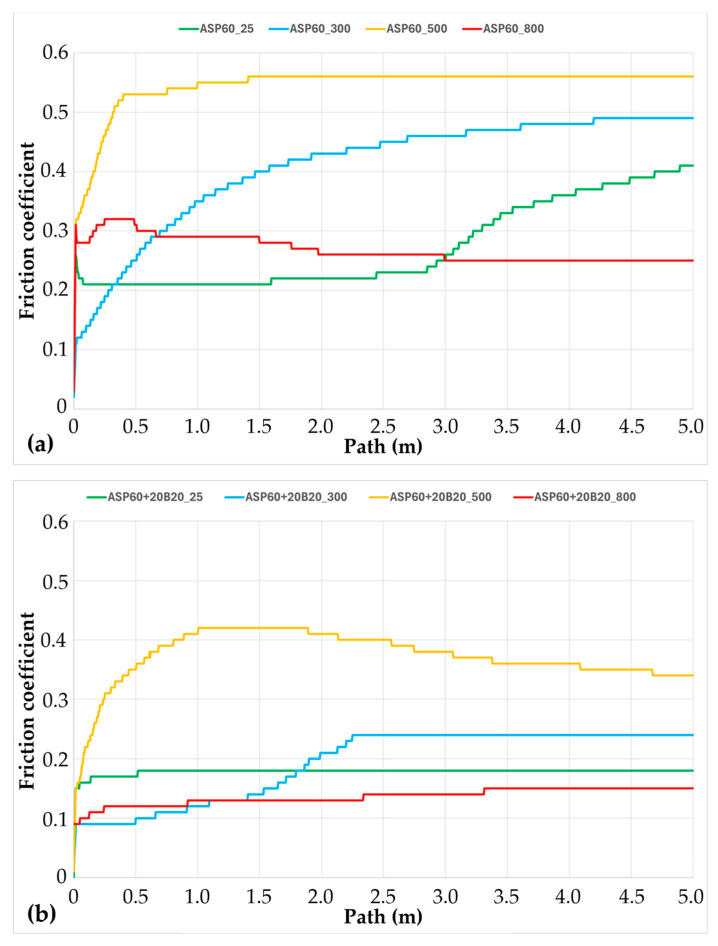
Evolution of friction coefficients during the ball-on-disc testing at laboratory temperature and 300, 500, and 800 °C of (**a**) ASP60 alloy; (**b**) ASP60 + CuSn20 alloy.

The temperature dependence of the coefficient of friction was assessed using a ball-on-disc tribological test, and the results are presented in Figure 19. The data reveal how frictional behavior varies with temperature across the different tested materials, providing valuable insight into their suitability for use in stone-cutting tool applications. These findings are particularly relevant given the operational requirements of such tools, where maintaining a low coefficient of friction is essential for minimizing heat generation and ensuring thermal stability during cutting. The observed trends contribute to a better understanding of the tribological performance of the developed composites under conditions simulating real working environments.

As shown in Figure 19a, the coefficient of friction for ASP60 high-speed steel increases with temperatures up to 500 °C. This trend can be attributed to enhanced adhesive interactions between the sample surface and the counter-body (sliding ball), which become more pronounced at elevated temperatures. At 800 °C, however, the coefficient of friction reaches its lowest recorded value among all tested temperatures. This reduction is likely due to surface softening and the formation of oxide layers, which reduce adhesion and thus lower the frictional forces during sliding.

Another significant factor influencing the tribological behavior at high temperatures is the microstructural evolution of the material. ASP60 is known to undergo over-tempering at temperatures in the range of 540–570 °C, leading to microstructural coarsening. At 800 °C, this effect is likely exacerbated, contributing to a softening of the wear track. The continuous decrease in the friction coefficient during testing may also result from progressive heating of the contact area, further reducing the resistance to sliding.

At room temperature (25 °C), the friction coefficient initially remains stable but increases toward the end of the test, suggesting the breakdown of thin surface films or oxide layers. This phenomenon occurs more rapidly at 300 °C, where the elevated temperature accelerates film degradation and increases adhesive wear. In contrast, at 500 °C, the friction coefficient rises sharply at the beginning of the test and remains nearly constant throughout the tribological cycle, indicating the formation of a stable, high-friction surface layer due to persistent adhesive interactions.

The incorporation of CuSn20 alloy into the ASP60 high-speed steel matrix resulted in a consistent reduction of the coefficient of friction across all tested temperatures (Figure 19b). This effect can be attributed to the solid lubricant characteristics of CuSn20, which include its capacity to form oxide layers that reduce adhesive forces at the contact interface, as well as its inherent resistance to welding under load. This friction-reducing behavior is particularly advantageous, as it leads to lower heat generation during tribologically demanding operations, thereby enhancing the thermal stability of the cutting tool system and further extending the total lifespan of diamond-reinforced tools with the ASP60 matrix.

Tribological testing at 25 °C and 300 °C revealed a gradual increase in the coefficient of friction during the initial stages, followed by stabilization for the remainder of the test. At 500 °C, a different trend was observed: the coefficient of friction initially increased but then decreased, likely due to early-stage formation of protective oxide layers formed after sufficient sliding distance. At 800 °C, a notable reduction in the friction coefficient was recorded. This behavior may be associated with the partial melting of the CuSn20 alloy, which undergoes a peritectic reaction at approximately 798 °C. Localized frictional heating within the wear track may further elevate the temperature, increasing the volume fraction of molten CuSn20 and further reducing friction. It is important to note that such extreme temperatures represent atypical or critical operating conditions, as cutting tools are typically water-cooled in practical applications.

In addition to the favorable tribological properties, all tested materials exhibited a zero measurable wear rate under the applied conditions. This observation aligns with the well-established reputation of ASP60 as a high-wear-resistance material. Despite some visible changes in the wear track—likely associated with the formation of a plastically deformed surface layer—no significant material loss could be detected using profilometric analysis. This exceptional wear resistance is a highly desirable property for prolonging the service life of tools subjected to intensive mechanical and thermal loading.

In light of the presented findings, the tribological performance of the ASP60 + CuSn20 + 20C composite—comprising TiC-coated diamond grits—was not assessed using standard ball-on-disc testing methods. This decision was made on the basis that the incorporation of extremely hard diamond reinforcements would significantly alter the contact mechanics, with the diamond particles acting as gliding bodies during sliding. Such behavior would likely suppress meaningful interaction between the counter-body and the metallic matrix, thereby limiting the relevance and interpretability of conventional tribological data obtained by the current testing equipment.

Nevertheless, the exceptionally low coefficients of friction and the absence of measurable wear observed in the ASP60 + CuSn20 matrix indicate that the diamond-reinforced composite possesses substantial potential for applications subjected to extreme mechanical and tribological stresses, while also maintaining favorable thermal stability. The synergistic combination of superior wear resistance, minimal frictional heat generation, and the intrinsic hardness of diamond makes this material an excellent candidate for advanced cutting tools, particularly for low-speed sawing in quarry operations and other abrasive stone-processing environments.

## 5. Conclusions

The present manuscript documents the successful use of CuSn20 alloy to reduce deleterious porosity during the compaction of ASP60 high-speed steel via uniaxial hot pressing. The addition of CuSn20 not only reduced porosity, thereby improving mechanical properties, but also decreased friction coefficients across the tested temperature range. This effect is primarily attributed to the self-lubricating capabilities of CuSn20, which, upon exceeding 800 °C, further reduced friction due to its localized melting. Additionally, the ASP60 alloy exhibited no measurable wear rate, mainly due to the deformation-induced transformation of retained austenite into martensite, reinforced by various carbides, making the material highly wear-resistant. Optimal compaction conditions were identified at 1050 °C for 3 min, resulting in a dramatic porosity reduction to approximately 3.7%, as confirmed by both Archimedes and dimensional analysis. Mechanical properties, including hardness and bending strength, increased with compaction temperature, reaching their maximum at the highest tested temperature. Under these optimal conditions, the incorporation of TiC-coated diamond particles (c = 20) did not adversely affect mechanical properties. However, the compaction process, limited to a pressure of 35 MPa, resulted in a heterogeneous pore distribution, negatively impacting overall performance. Nevertheless, the study demonstrates that CuSn20 effectively reduces porosity by promoting particle rearrangement and gap filling, while also enhancing the tribological performance of ASP60-based composites. The composition of the developed tool materials indicates potentially higher application temperatures (under limited cooling conditions) compared to the currently known commercial matrices, which can operate at temperatures up to about 750 °C.

## Figures and Tables

**Table 1:** Chemical composition of the powdered ASP60 steel determined by XRF analysis.

Chemical Composition (wt.%)									
Cr	W	Mo	Co	V	Si	Mn	Ni	S	Fe
4.105 ± 0.06	7.270 ± 0.08	7.020 ± 0.08	11.144 ± 0.09	8.250 ± 0.08	0.407 ± 0.02	0.110 ± 0.01	0.112 ± 0.01	-	bal.

**Table 2:** Chemical composition of the powdered CuSn20 alloy determined by XRF analysis.

Chemical Composition (wt.%)			
Cu	Sn	Si	P
80.587 ± 0.100	19.314 ± 0.100	0.028 ± 0.005	0.071 ± 0.008

**Table 3:** Chemical composition of the ASP60 high-speed steel determined by elemental analysis.

Scheme	Chemical Composition (wt.%)		
	C	N	O
Prior DSC analysis	2.21 ± 0.066	0.046 ± 0.002	0.285 ± 0.028
Post DSC analysis	2.09 ± 0.062	0.047 ± 0.002	0.120 ± 0.011

**Table 4:** Rheological properties of ASP60 and CuSn20 powders determined by apparent density measurements (±*value corresponds to the relative standard deviation*).

Alloy	Measurement No.	Appar. Density (g/cm^3^)	Result (g/cm^3^)
ASP60	1	1.248	1.270 ± 0.052
	2	1.290	
	3	1.272	
CuSn20	1	3.175	3.183 ± 0.021
	2	3.183	
	3	3.192	

**Table 5:** Rheological properties of ASP60 and CuSn20 powders determined by tap density measurements (±*value corresponds to the relative standard deviation*).

Alloy	Measurement No.	Density_TAP_ (g/cm^3^)	Result (g/cm^3^)
ASP60	1	1.923	1.949 ± 0.110
	2	2.000	
	3	1.923	
CuSn20	1	4.237	4.225 ± 0.051
	2	4.237	
	3	4.202	

**Table 6:** Results of sample porosity determined by the Archimedes method and dimensional analysis.

Material	Compaction Temperature (°C)	Porosity Archimedes (%)	Porosity Dimensional (%)
ASP60	950	30.01 ± 0.09	28.66 ± 0.40
ASP60	1000	13.40 ± 0.04	11.56 ± 0.36
ASP60	1050	5.79 ± 0.01	8.31 ± 0.27
ASP60 + CuSn20	950	7.96 ± 0.02	10.21 ± 0.33
ASP60 + CuSn20	1000	3.88 ± 0.01	4.25 ± 0.61
ASP60 + CuSn20	1050	3.70 ± 0.01	3.69 ± 0.57

**Table 7:** Results of bending tests showing the average flexural strength of the materials.

Material	Compaction Temperature (°C)	σ_bending_ (MPa)
ASP60	950	81.8 ± 1.8
ASP60	1000	249.6 ± 20.8
ASP60	1050	267.3 ± 26.4
ASP60 + CuSn20	950	260.5 ± 3.7
ASP60 + CuSn20	1000	321.3 ± 17.6
ASP60 + CuSn20	1050	374.5 ± 36.7

**Table 8:** Vickers (HV30) hardness of the prepared specimens measured on the tensile and compressive sites, accompanied by a relative standard deviation (RSD) and a 95% confidence interval.

		Porous Site			Crack Site		
Material	Compaction Temperature (°C)	Average HV30	RSD	Confidence	Average HV30	RSD	Confidence
ASP60	1000	695.7	85.8	106.6	641.9	50.1	62.3
ASP60	1050	888.7	72.7	90.2	854.1	32.5	40.4
ASP60 + CuSn20	1000	832.7	20.6	25.5	852.7	30.2	37.6
ASP60 + CuSn20	1050	794.0	13.2	16.4	906.0	18.5	23.0

**Table 9:** Rockwell (HRC) hardness of the prepared specimens measured on the tensile and compressive sites, accompanied by a relative standard deviation (RSD) and a 95% confidence interval.

		Porous Site			Crack Site		
Material	Compaction Temperature (°C)	Average HRC	RSD	Confidence	Average HRC	RSD	Confidence
ASP60	1000	59.4	2.7	3.4	56.0	3.5	4.4
ASP60	1050	62.0	2.3	2.9	61.0	1.2	1.5
ASP60 + CuSn20	1000	64.4	0.5	0.7	64.8	0.4	0.6
ASP60 + CuSn20	1050	62.6	0.5	0.7	64.8	0.4	0.6

**Table 10:** Results of bending tests show the average flexural strength of the composite.

Material	Compaction Temperature (°C)	σ_bending_ (MPa)
ASP60 + CuSn20 + 20C	1050	359.1 ± 26.5

## Data Availability

The original contributions presented in this study are included in the article. Further inquiries can be directed to the corresponding author.
